# Enhancing POLST accessibly to achieve Age Friendly Health System (AFHS) recognition in Long Term Care: What Matters

**DOI:** 10.1093/geroni/igaf122.3160

**Published:** 2025-12-31

**Authors:** Kevin Overbeck, Elyse Perweiler, Jennifer DeGennaro, Cindy Nolan

**Affiliations:** Rowan-Virtua School of Osteopathic Medicine, Stratford, New Jersey, United States; Rowan-Virtua School of Osteopathic Medicine, Stratford, New Jersey, United States; Rowan-Virtua School of Osteopathic Medicine, Stratford, New Jersey, United States; Rowan-Virtua School of Osteopathic Medicine, Stratford, New Jersey, United States

## Abstract

The NJ POLST document addresses what matters most to older individuals through its “Goals of Care” section ensuring that patient priorities are clearly communicated. As part of the pursuit of Age Friendly Health System (AFHS) recognition, a nursing home utilizing PointClickCare (PCC) as its EMR aimed to address the availability of the NJ POLST forms. While POLST documents are consistently scanned into PCC, they were not readily accessible to acute care teams in the regions three (3) leading hospital systems which use Epic EMR. This gap in accessibility posed a critical barrier to seamless, patient-centered care transitions. An interprofessional team reviewed baseline metrics as well as the current workflows for scanning and storing NJ POLST forms in PCC. Collaborative efforts with hospital system representatives explored technical solutions for integrating POLST documents into Epic. Metrics tracked included the number of residents with completed POLST forms, the proportion of forms successfully transferred to Epic, and the time required to complete this transfer process. 25% of fifty (50) long-term care residents in the nursing home had a completed NJ POLST document accessible in PCC. Prior to implementation 5% had the NJ POLST document electronically accessible in the Epic system of partnering hospitals and 100% had this document accessible upon project completion. This approach can serve as a model for other nursing homes pursuing AFHS recognition. The project demonstrated the feasibility of improving NJ POLST document accessibility across EMR platforms ensuring that patient preferences are available to acute care teams when it matters most.

